# Proteomics Identified UDP-Glycosyltransferase Family Members as Pro-Viral Factors for Turnip Mosaic Virus Infection in *Nicotiana benthamiana*

**DOI:** 10.3390/v15061401

**Published:** 2023-06-20

**Authors:** Kaida Ding, Zhaoxing Jia, Penghuan Rui, Xinxin Fang, Hongying Zheng, Jianping Chen, Fei Yan, Guanwei Wu

**Affiliations:** 1 State Key Laboratory for Managing Biotic and Chemical Threats to the Quality and Safety of Agroproducts, Institute of Plant Virology, Ningbo University, Ningbo 315211, China; 13634094975@163.com (K.D.); wwwjiazhaoxing@126.com (Z.J.); 15755308130@163.com (P.R.); 18858407400@163.com (X.F.); zhenghongying@nbu.edu.cn (H.Z.); chenjianping1@nbu.edu.cn (J.C.); 2 Key Laboratory of Biotechnology in Plant Protection of MARA and Zhejiang Province, Institute of Plant Virology, Ningbo University, Ningbo 315211, China

**Keywords:** proteomics, turnip mosaic virus, viral replication, *Nicotiana benthamiana*, uridine diphosphate-glycosyltransferase (UGT), reactive oxygen species

## Abstract

Viruses encounter numerous host factors that facilitate or suppress viral infection. Although some host factors manipulated by viruses were uncovered, we have limited knowledge of the pathways hijacked to promote viral replication and activate host defense responses. Turnip mosaic virus (TuMV) is one of the most prevalent viral pathogens in many regions of the world. Here, we employed an isobaric tag for relative and absolute quantitation (iTRAQ)-based proteomics approach to characterize cellular protein changes in the early stages of infection of *Nicotiana benthamiana* by wild type and replication-defective TuMV. A total of 225 differentially accumulated proteins (DAPs) were identified (182 increased and 43 decreased). Bioinformatics analysis showed that a few biological pathways were associated with TuMV infection. Four upregulated DAPs belonging to uridine diphosphate-glycosyltransferase (UGT) family members were validated by their mRNA expression profiles and their effects on TuMV infection. NbUGT91C1 or NbUGT74F1 knockdown impaired TuMV replication and increased reactive oxygen species production, whereas overexpression of either promoted TuMV replication. Overall, this comparative proteomics analysis delineates the cellular protein changes during early TuMV infection and provides new insights into the role of UGTs in the context of plant viral infection.

## 1. Introduction

During infection, a complicated arms race takes place wherein plant viruses must simultaneously rewire cellular pathways they require for replication while evading the host cell’s defenses. The integration of proteomic methods with virology yielded significant insights into mechanisms of virus replication, antiviral host responses and viral subversion of host defenses. Host proteins that are upregulated and downregulated during virus infection can be key plant proteins and resistance factors that the virus utilizes or evades, respectively, to promote viral replication. Isobaric tags for relative and absolute quantitation (iTRAQ) is a technology that utilizes isobaric reagents to label the primary amines of peptides and proteins to study quantitative changes in the proteome by tandem mass spectrometry. Currently, the iTRAQ methodology is one of the major quantitation tools used in differential plant proteomic research [1].

Uridine diphosphate-glycosyltransferases (UGTs) are a superfamily of enzymes that transfer a glycosyl moiety from uridine diphosphate (UDP) sugars to acceptor molecules in plants, animals, fungi, bacteria and viruses [2]. Glycosylation can affect the stability, transport, storage, reactivity and bioactivity of the sugar acceptors [3]. In plants, UGTs can glycosylate a variety of metabolites at O-, N-, S- and C-atoms, and lead to a high glycodiversity of specialized metabolites. Plant UGTs not only play critical roles in adaptation to various environments but also endow plant natural products with great pharmaceutical and ecological significance. In plants, the glycosylated products play important roles in hormone homeostasis, development, defense and abiotic stress [4,5,6,7,8,9]. UGTs are generally promiscuous in their use of acceptors, making it challenging to reveal the function of UGTs in vivo. Recently, researchers developed bioinformatic and experimental methods to predict or characterize plant UGTs [10,11]. They constructed a comprehensive plant UGT database, which contains 285,293 plant UGTs from 2858 plants, nearly nine times more than those in the published literature and database collections; however, only 0.1% UGTs were functionally studied until now [4,10]. There was little in-depth investigation into the role of these UGTs in plant–viral interactions.

Potyviruses are the largest group of known plant RNA viruses and include many agriculturally important viruses, include plum pox virus, soybean mosaic virus, turnip mosaic virus (TuMV) and potato virus Y [12,13]. Because of their importance, potyviruses were more studied than many other viruses [12,14,15,16]. Potyviruses have a genome that is a single-stranded, positive-sense RNA of ∼10,000 nucleotides (nt). The genome has one major open reading frame (ORF), which is translated into one large polyprotein, and a small overlapping ORF. The long polyprotein is proteolytically processed by three viral proteases into 10 mature proteins and a variety of intermediate precursors [14]. Among potyviruses, TuMV is of particular scientific interest as it has the broadest host range of any of the potyviruses, infects not only the model species Arabidopsis thaliana and *Nicotiana benthamiana* but also many agriculturally and scientifically important dicotyledonous and monocotyledonous plants species [17]. Understanding the underlying biology of viral infection, more specifically the host proteins and cellular processes that are essential for viral infection and replication, will identify targets for development of novel host-directed therapies.

In this work, we used iTRAQ to investigate the proteome changes in the host in response to infection by wild type and replication-defective TuMV isolates. The iTRAQ quantification results, followed by KEGG pathway and GO analyses, provided comprehensive data on responses of tobacco host proteins upon TuMV infection during the early infection stage. We further validated the differentially accumulated UDP-glycosyltransferase family members and identified two of them, UGT74F1 and UGT91C1, that have pro-viral roles in TuMV infection.

## 2. Materials and Methods

### 2.1. Plant Materials and Virus Inoculation

*Nicotiana benthamiana* plants were grown in a climate-controlled growth chamber at 22 °C in a 16 h light/8 h dark cycle, with a relative humidity of 60%. Then, 3- to 4-week-old *N. benthamiana* plants were used for *Agrobacterium tumefaciens* (strain GV3101)-mediated transient expression. For viral infection analysis, Agrobacterium cultures carrying TuMV-GFP or TuMV-GFP//△GDD infectious clones were infiltrated into *N. benthamiana* leaves. Each plant was grown in an individual pot to avoid potential cross contamination. For each biological replicate, local infected leaves from three TuMV-GFP-infected or TuMV-GFP//△GDD-infected *N. benthamiana* plants were collected and combined as one sample at 3 dpi. Three biological replicates of samples were collected.

### 2.2. Protein Extraction, Trypsin Digestion, TMT/iTRAQ Labelling and LC-MS/MS Analysis

Frozen leaf samples (approximately 1.0 g for each replicate) were ground to a fine powder using liquid nitrogen, and were then lysed ultrasonically. An equal volume of Tris-phenol was added, and the samples were centrifuged at 12,000× *g* for 10 min at 4 °C. Proteins were precipitated, washed with methanol and acetone, and the final pellet was resuspended in 1% SDS. Protein concentrations were determined and then digested according to a previously described procedure [18]. LC-MS was performed using the Q Exactive TM Plus Orbitrap Mass Spectrometer (Thermo Fisher Scientific, Rockford, IL, USA) and the Easy-nLCTM 1200 system (Thermo Fisher Scientific). Parameters were set as follows: electrospray voltage, 2.1 kV; automatic gain control (AGC), 5E4; survey scans were acquired at a resolution of 1,200,000; resolution for HCD spectra, 15,000.

### 2.3. Database Search and Bioinformatic Analysis

The acquired MS/MS data were processed using the MaxQuant search engine (v. 1.5.2.8) for both protein identifications and iTRAQ quantitation against the *Nicotiana benthamiana*_4100 database. The false discovery rate (FDR) was adjusted to 1%. Peptides and protein identification were both filtered by an FDR of 1%, and each identified protein was required to have at least two distinct peptides. For this study, strict unused confidence score > 1.3 (equivalent to 95% confidence level) was used for significant identification of proteins. Protein lists were interpreted according to fold change in expression. The cut-off for high abundance (>1.3-fold over normal, *p* < 0.005) and low abundance (<0.67-fold over normal, *p* < 0.05) proteins were selected to identify differentially abundant proteins (DAPs).

To determine the biological and functional properties of the differentially expressed proteins (DEPs), gene ontology (GO) annotations based on biological process, cellular component and molecular function were derived from the UniProt-GOA database and InterProScan software. KEGG pathway annotation was carried out using the Kyoto Encyclopedia of Genes and Genomes database.

### 2.4. RNA Extraction and RT-qPCR

Total RNA extraction from *N. benthamiana* tissues, synthesis of the first-strand cDNA, PCR and qPCR was performed essentially as described previously [19]. To determine the specificity of each reaction, a melting curve analysis was performed. The relative virus or gene concentration was calculated using the 2^−ΔΔCT^ method with Actin II as the reference gene [20]. All primers used in this study are listed in Table S1.

### 2.5. Gene Cloning and Plasmid Construction

PrimeSTAR GXL DNA Polymerase (Takara, Japan) was used to amplify all coding sequences of *NbUGTs* using the primers listed in Table S1, and Gateway technology (Thermo Fisher Scientific, USA) was employed for plasmid construction. To create the *N. benthamiana* UGT VIGS constructs, a 300-nt gene-specific cDNA fragment was amplified. The PCR amplicon was cloned into TRV2:Lic as described previously [21], resulting in the vectors pTRV2::NbUGTs. All constructs were verified by sequencing.

### 2.6. Western Blotting

*N. benthamiana* leaf tissues were ground into fine powder in liquid nitrogen, and lysed with cell lysis buffer as previously described [19]. Protein samples were separated by SDS-PAGE and transferred to polyvinylidene difluoride (PVDF) membranes. After blocking with 5% milk-Tris-buffered saline Tween 20 (TBST), the membrane was incubated with the primary antibodies against the indicated target proteins and corresponding horseradish peroxidase (HRP)-conjugated secondary antibodies. Chemiluminescent imaging was performed using an Immobilon Western chemiluminescent horseradish peroxidase (HRP) substrate (Millipore) according to the manufacturer’s instructions.

## 3. Results

### 3.1. Wild Type and Replication-Deficient TuMV Infection

**Figure 1:**
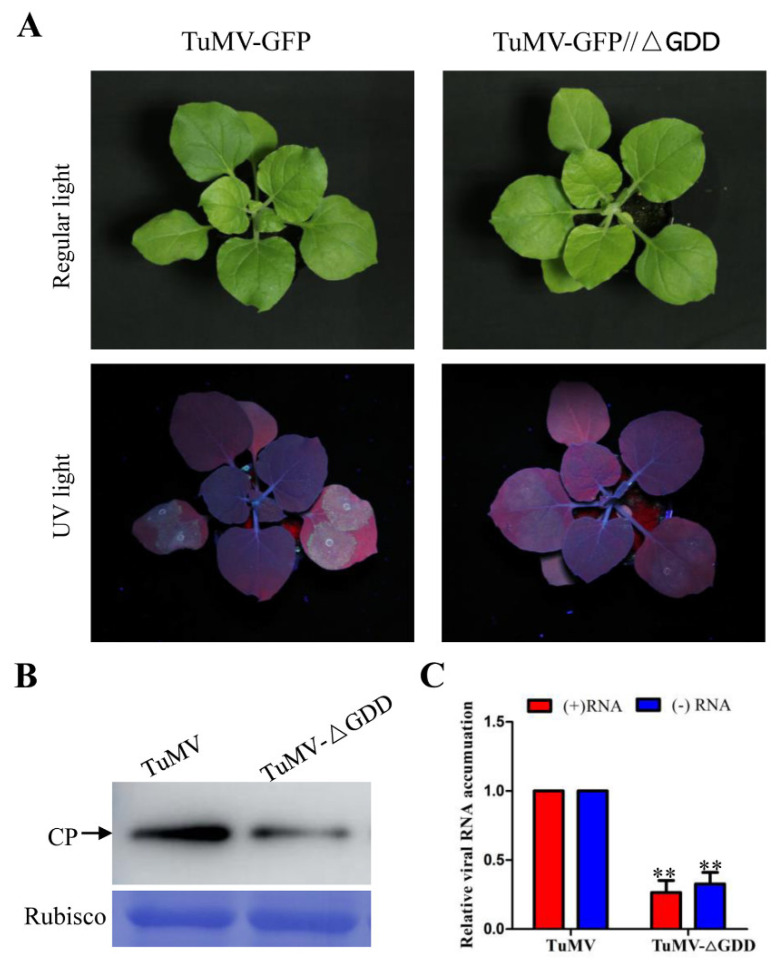
Infectivity test of TuMV-GFP and replication-deficient TuMV-GFP//△GDD in *N. benthamiana*. (**A**) Representative photographs of TuMV-GFP and TuMV-GFP//△GDD infection in *N. benthamiana* plants. Pictures were taken under regular light and UV light at 4 days post infection. (**B**) Western blot detection of TuMV coat protein accumulation in the inoculated leaves. (**C**) RT-qPCR results showing viral positive-sense (+) and negative-sense (−) RNA levels in TuMV-GFP- and TuMV-GFP//△GDD-infected local leaves. The error bar represents the standard deviation of three biological replicates of a representative experiment. Statistical analysis was performed using Student’s *t*-test (**, *p* < 0.01).

To explore the host factors involved in host defense and TuMV replication, wild-type TuMV and a replication-deficient TuMV mutant were employed in this study. A full-length CDNA clone of TuMV UK-1 isolate was described previously [22]. We also used a replication-defective mutant, designated as TuMV-△GDD, which has a deletion in the coding sequence for the GDD (glycine-aspartic acid-aspartic acid) motif that is required for the activation of the RNA-dependent RNA polymerase (NIb) [23]. TuMV-GFP and TuMV-GFP//△GDD were agro-infiltrated into *N. benthamiana* leaves at an OD600 of 0.1. At 3 days post agro-infiltration infection (dpai) with TuMV-GFP, there was bright fluorescence in inoculated leaves under UV light, while the fluorescence was less with TuMV-GFP//△GDD (Figure 1A). Western blotting assay against the levels of CP accumulation in the inoculated leaves gave similar results (Figure 1B). The inoculated tissues were collected at 60 h post-agroinoculation and qPCR was used to analyze the accumulation of positive-sense (+) and negative-sense (−) viral RNA. TuMV infects and replicates in the primarily infected cells within this time window, and viral intercellular movement usually does not occur until 4 dpai [24]. The RT-qPCR results showed that the level of both positive-sense and negative-sense viral RNA accumulation resulting from the TuMV-△GDD was significantly less than in the wild type TuMV. The small amount of CP or viral RNA accumulation of △GDD resulted from the activity of the 35S promoter.

### 3.2. Identification and Quantification of Total Proteins by iTRAQ

**Figure 2:**
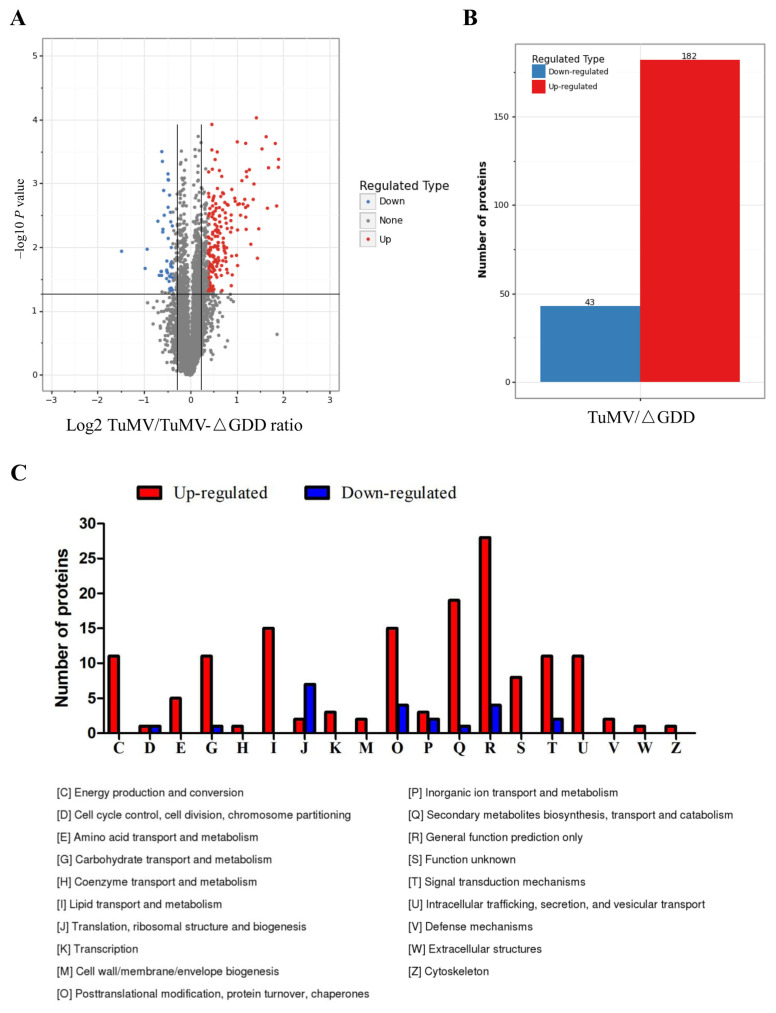
Number and KOG functional classification of differentially abundant host proteins between TuMV-GFP- and TuMV-GFP//△GDD-infected *N. benthamiana* leaves. (**A**) Volcano plot illustrating the significantly differentially accumulated proteins. The –log10 (Benjamini-Hochberg corrected *p* value) is plotted against the log2 (fold change: TuMV/TuMV-△GDD). The non-axial vertical lines denote ±1.3-fold change while the non-axial horizontal line denotes *p* = 0.05, which is our significance threshold (prior to logarithmic transformation). (**B**) Histogram displaying the number of differentially abundant proteins within a specific range of fold changes. (**C**) GO enrichment classification of differential accumulated proteins. Red colour bars indicate upregulated proteins and blue colour bars indicate downregulated proteins.

Inoculated tissues were subjected to iTRAQ analysis. A total of 52,117 spectra (22,618 distinct spectra) were obtained from reverse phase HPLC and LC-MS/MS proteomic analysis. After strict cut-off for unused protein score > 1.3 (95% confidence) with 1% FDR, 7632 proteins were identified. Differentially accumulated proteins (DAPs) were selected with significant changes (*p*-value < 0.05), with cut-off points fixed at >1.3-fold change (*p*-value < 0.05) for increased abundance and <0.77-fold change (*p*-value < 0.05) for proteins with reduced abundance (Figure 2A, Table S2). A total of 225 DAPs were identified, 182 proteins with increased abundance and 43 proteins with reduced abundance (Figure 2B).

### 3.3. GO Enrichment Analyses of Host DAPs

GO enrichment classification revealed that these DAPS were associated with a range of biological process, molecular functions and cellular component categories. The most strongly affected biological processes were those involved in RNA metabolic, macromolecule biosynthesis and gene expression (Figure S1), suggesting that TuMV requires host transcriptional and translational machinery for generation of viral proteins. The most strongly affected cellular components were extracellular region, clathrin-coated vesicles and intracellular vesicles (Figure S1). Moreover, the top three most affected pathways were involved in mitogen-activated protein kinase cascade (MAPK) signalling, plant-pathogen interactions and phenylpropanoid biosynthesis (Figure S1). These DAPS were classified into 19 groups based on their function by KOG annotation. Most of up-regulated proteins were involved in secondary metabolite biosynthesis, lipid metabolism, posttranslational modification and intracellular trafficking, whereas most downregulated proteins were involved in ribosomal structure and biogenesis and protein turnover (Figure 2C).

### 3.4. Validation of UDP- Glycosyltransferases (UGTs) Proteins Using RT-qPCR

**Figure 3:**
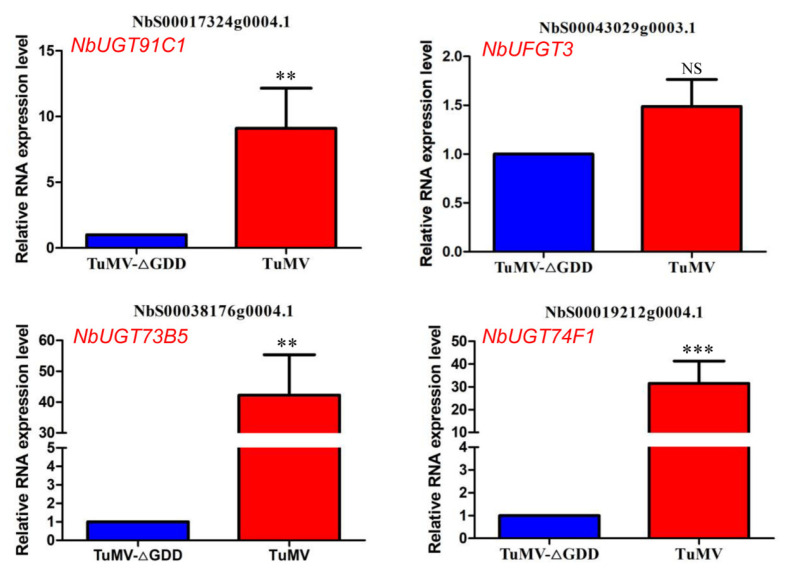
Validation of the selected NbUGTs RNA expression in the leaves of TuMV-GFP- and TuMV-GFP//△GDD-infected *N. benthamiana* plants by real time RT-PCR. ** and *** represent statistically significant differences by the Student *t*-test between groups at *p* < 0.01 and *p* < 0.001, respectively. NS, not significant.

The phenylpropanoid biosynthesis pathway in plants is responsible for the biosynthesis of a huge number of secondary metabolites. The diversity of these molecules can be further increased by the action of UGTs [25]. Interestingly, we found four UGTs among the significantly up-regulated host DAPs: NbS00038176g0004.1 (NbUGT73B5), NbS00019212g0004.1 (NbUGT74F1), NbS00017324g0004.1 (NbUGT91C1) and NbS00043029g0003.1 (NbUFGT3). We used RT-qPCR to validate these four DAPs at the transcriptome level. The results showed that mRNA levels of all these four NbUGTs, except for UFGT3, were significantly increased under TuMV-GFP infection compared with TuMV-GFP//△GDD (Figure 3). We selected NbUGT73B5, NbUGT74F1 and NbUGT91C1 for further study.

### 3.5. Virus-Induced Gene Silencing Screen Revealed That NbUGT91C1 and NbUGT74F1 Are Required for TuMV Infection

**Figure 4:**
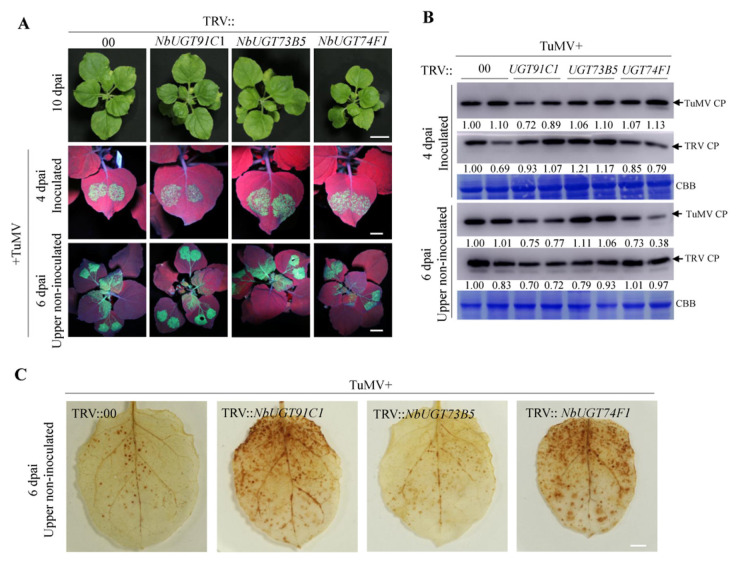
Effect of silencing the selected NbUGTS on TuMV infection in *N. benthamiana* plants. (**A**) Phenotypes of *N. benthamiana* with silenced NbUGTS before and after TuMV infection (top row). The empty vector TRV::00 was used as control. Lower panels show GFP fluorescence resulting from TuMV-GFP infection under a hand-held UV lamp. (**B**) TuMV and TRV CP accumulation levels in the TRV+TuMV-inoculated plants. Both inoculated and upper non-inoculated tissues were harvested for Western blotting assay. (**C**) The oxidative burst in the upper NbUGTs-silenced and control leaves observed by staining with diaminobenzidine (DAB) at 6 dpai after TuMV infection. Bars, 5 cm.

To identify the role of these NbUGTs in TuMV infection, a virus-induced gene silencing assay using tobacco rattle virus (TRV) was performed on *N. benthamiana*. Two-week-old plants were agro-inoculated with an infectious clone of tobacco rattle virus (TRV) harbouring each NbUGT gene-specific fragment designed by using the Sol Genomics Network VIGS tool [26]. A wild-type TRV clone without an additional gene fragment inserted, designated as TRV::00, served as the control. At 10 dpai, VIGS had spread systemically based on the appearance of photobleaching in noninfected tissues as a result of silencing of the gene phytoene desaturase (TRV::PDS). Gene silencing efficiency of each of the NbUGTs was determined by RT-qPCR (Figure S2). The phenotypes of TRV::NbUGTs-infected plants did not differ substantially from the TRV::00 control, except for TRV2::NbUGT74F1, which displayed growth inhibition (Figure 4A).

TuMV was then agro-inoculated onto the silenced leaves of each treatment as a second virus. At 4 dpai, most plants infected with TRV::*NbUGT*s had comparable GFP fluorescence intensity under UV illumination to the TRV:00 controls, except for TRV::*NbUGT91C1*, which appeared to have weaker fluorescence (Figure 4A). This result was confirmed by Western blotting assay (Figure 4B). We also found that silencing of NbUGT73B5 slightly increased TRV CP accumulation (~1.2 fold), while silencing of the other two tested NbUGTs did not affect TRV CP accumulation compared to the TRV::00 control (Figure 4B). Then, we evaluated the effect of silencing UGTs on TuMV infection in upper non-inoculated leaves at 6 dpai. TRV::*NbUGT74F1* and TRV::*NbUGT91C1* plants showed a remarkably weaker intensity of GFP fluorescence than TRV::00 (Figure 4A). A Western blotting assay against the levels of TuMV CP accumulation gave similar results, with lower levels (~75%) in TRV::*NbUGT74F1* and TRV::*NbUGT91C1* plants than in the TRV:00 plants (Figure 4B). TRV CP accumulation was similar among TRV::00, TRV::*NbUGT74F1* and TRV::*NbUGT73B5* plants, but much less in TRV::*NbUGT91C1* plants (~70%) (Figure 4B). Taken together, these results suggest that NbUGT74F1 and UGT91C1 may participate in TuMV infection.

To exclude the effect of TRV infection on the second virus infection assay, and further confirm the role of silencing NbUGT91C1 or NbUGT74F1 on TuMV infection, the intron-spliced hairpin RNA-mediated RNA interference method was used to downregulate the expression of NbUGT91C1 or NbUGT74F1 in *N. benthamiana* plants. The artificial RNA silencing constructs expressing partial β-glucuronase hairpin RNA (dsGUS) or NbUGT hairpin RNA (dsNbUGT) were transiently expressed in the same leaves of *N. benthamiana*. TuMV-GFP was then inoculated at 1 dpai. The green fluorescence in dsNbUGT74F1- or dsNbUGT91C1-treated regions was much weaker than that in dsGUS at 3 dpai (Figure S3A). RT-qPCR results indicated that the expression level of NbUGT in the dsNbUGT-treated region decreased to 20–30% of that in the control dsGUS (Figure S3B). TuMV RNA accumulation was significantly less in dsNbUGT-treated leaves than in dsGUS-treated leaves (Figure S3B). Western blot analysis also showed that the accumulation of TuMV CP in dsNbUGT-treated leaves was decreased to 40–60% compared with dsGUS (Figure S3C). These combined results indicate that NbUGT91C1 and NbUGT74F1 are required for TuMV infection.

To further examine the contribution of NbUGT91C1 and NbUGT74F1 in hydrogen peroxide (H_2_O_2_) production during TuMV infection, the infected systemic leaves on the silenced and control plants were stained with diaminobenzidine (DAB) at 6 dpi with TuMV. The NbUGT91C1- and NbUGT74F1-silenced plants showed strong, brown precipitates, compared with the control and NbUGT73B5-silenced plants (Figure 4C). These data suggest that silencing of NbUGT91C1 or NbUGT74F1 induces reactive oxygen species (ROS) production to suppress TuMV infection.

### 3.6. Over-Expression of NbUGT74F1 or NbUGT91C1 Promotes TuMV Replication

**Figure 5:**
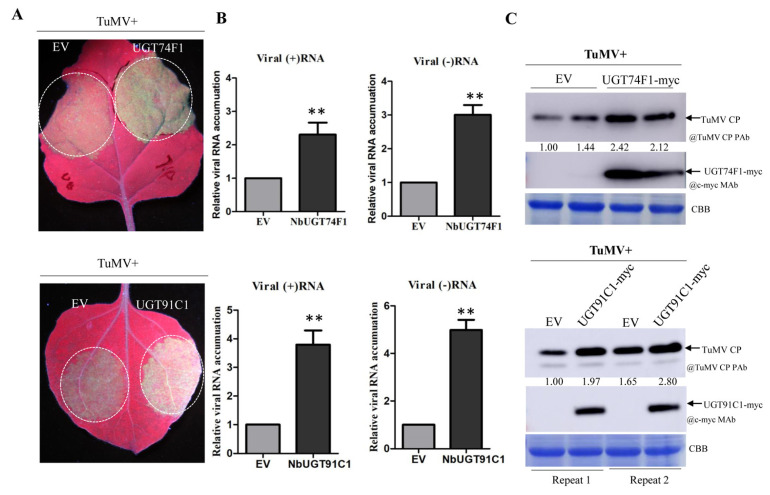
Effect of NbUGT74F1 and NbUGT91C1 overexpression on TuMV infection. (**A**) GFP fluorescence in *N. benthamiana* plants inoculated with TuMV-GFP together with GUS (control), NbUGT74F1, or NbUGT91C1. Plants were photographed under a hand-held UV lamp at 3 dpai. (**B**) RT-qPCR results showing the quantification of positive-strand viral genomic RNA [(+)RNA] or negative-strand viral genomic RNA [(−) RNA] accumulation in *N. benthamiana* plants agroinfiltrated with different combinations of plasmids from (**A**). The infiltrated leaf tissues were collected for RNA purification at 65 h post agroinfiltration (hpai) and RT-qPCR was performed with TuMV nib-specific primers using the actin II transcript level as an internal control. The error bar represents the standard deviation of three biological replicates of a representative experiment. Statistical analysis was performed using Student’s *t*-test **, *p* < 0.01). (**C**) Western blotting analysis of the TuMV coat protein (CP) in the infiltrated leaf tissues from *N. benthamiana* plants in (**A**) at 65 hpai. Coomassie Brilliant Blue R-250-stained RuBisco large subunit serves as a loading control. TuMV CP was detected with anti-TuMV CP polyclonal antibody. NbUGT74F1 and NbUGT91C1 were detected with anti-c-Myc monoclonal antibodies.

We then transiently co-expressed NbUDP74F1 or NbUGT91C1 with TuMV-GFP in *N. benthamiana* leaves and monitored the viral infection progress by measuring both RNA and protein accumulation levels. Under UV light, TuMV-GFP fluorescence was brighter in the leaf tissues co-infiltrated with TuMV::GFP and NbUDP74F1 or NbUGT91C1, compared with that in the control agroinfiltrated with TuMV-GFP and empty vector (Figure 5A). An RT-qPCR assay of viral genomic RNA [either sense (+) or negative sense (−)] was further performed to evaluate viral replication levels at 65 h post agroinfiltration (hpai). Consistently, significantly higher levels of TuMV (+) or (−) RNA were detected in the leaf tissues agroinfiltrated with TuMV-GFP together with NbUGT74F1 or NbUGT91C1 at this time point, compared with that in the control (Figure 5B). Further Western blotting analysis against the viral CP accumulation level showed that about 2.0-fold stronger TuMV CP signals were detected in the leaf samples overexpressing NbUGT74F1 or NbUGT91C1 at 72hpai (Figure 5C). These results suggest that NbUGT74F1 and NbUGT91C1 have a pro-viral role in supporting TuMV replication.

## 4. Discussion

As obligate intracellular parasites, viruses employ a temporal cascade of protein expression and interactions with the host [27]. The last decade documented the increasing application of proteomic approaches to virology studies [28]. In recent years, iTRAQ was increasingly used for comparative proteomics in response to various biotic and abiotic stresses in plants [1,29,30,31]. Thus, we used iTRAQ technique to identify DAPs in wild-type and replication-defective TuMV-infected *Nicotiana benthamiana*. Our aim was to compare the proteins involved in defense response and viral replication using these two TuMV clones (Figure 1). We, therefore, compared the proteome profiles of TuMV- and TuMV-△GDD infected *N. benthamiana* plants at 3 dpi, a time at which TuMV intercellular movement does not usually take place [24].

Defense proteins induced by TuMV and TuMV-△GDD differed in abundance, and included a network of MAPK signalling, plant–pathogen interaction and phenylpropanoid biosynthesis (Figure 2 and Figure S2). Plants primarily detect approaching microbes and initiate immune signals via MAPK cascade and WRKY transcription factors. This then induces the production of immune molecules such as secondary metabolites and pathogenesis-related proteins [32]. The majority of defense molecules are finally secreted into pathogen-contacting extracellular space to terminate pathogenesis [33]. Consistently, the mostly strongly affected cellular components are extracellular region, clathrin-coated vesicles and intracellular vesicles (Figure S2). Vesicle trafficking plays important roles in plant immunity [34]. Clathrin-mediated endocytosis is a key process in vesicular trafficking that transports a wide range of cargo molecules from the cell surface to the interior [35]. Accumulated evidence suggests that TuMV also hijacks endocytic pathways for viral intracellular movement and replication [18,36,37]. Our proteomic data imply possible roles for these differential accumulated endocytic proteins in TuMV replication. Emerging evidence showed that extracellular vesicles play a prominent role in plant–microbe interactions by safely transporting functional molecules, such as proteins and RNAs to interacting organisms [38]. TuMV reorganizes the endomembrane system of the infected cell to generate endoplasmic reticulum-derived motile vesicles containing viral replication complexes, and the membrane-associated viral protein 6K2 plays a key role in the formation of these vesicles [39,40]. TuMV components can be released into the extracellular space by 6K2-induced vesicles [41], suggesting that our identified DAPs may function against TuMV in the extracellular region.

ROS homeostasis is maintained at a low level under normal growth conditions. Excess ROS accumulation leads to membrane oxidation and disruption of the photosystems, resulting in severe cellular damage [42]. Plant immune responses are generally heightened and may result in host cell death by elevated salicylic acid (SA) and ROS levels. SA is an essential plant defense hormone that promotes immunity against biotrophic and semi-biotrophic pathogens [43]. SA can undergo multiple chemical modifications, including hydroxylation, glycosylation, methylation and amino acid conjugation, which contributes to the dynamics of SA levels in plants and plays important roles in control of SA homeostasis [44]. Several UGTs were reported to participate in the glycosylation of SA, which can be involved in feedback regulation of SA biosynthesis [45,46]. In this study, we found four upregulated accumulated proteins belonging to UGT family members under TuMV infection (Table S2), and identified that the knock down of NbUGT74F1 caused growth inhibition and resistance to TuMV infection (Figure 4A). Arabidopsis UGT74F1 has high in vitro activity in converting SA to SA-*O*-β-d-glucoside (SAG) [47,48], and during pathogen infection, a large portion of the newly synthesized SA is converted to SAG. In atugt74f1 mutants, SAG levels are reduced, whereas free SA levels are elevated compared to wild-type plants [46]. Therefore, we proposed that knock down of NbUGT74F1 increases SA accumulation. Consistently, much more ROS accumulated in NbUGT74F1-silenced leaves (Figure 4C). NbUGT91C1 shows highest similarity with AtUGT79B1, encoding an anthocyanin 3-*O*-glucoside, which can convert cyanidin 3-*O*-glucoside to cyanidin 3-Oxylosyl (1–>2) glucoside [49]. Anthocyanins were shown to act as antioxidants to scavenge ROS in plants [50]. Anthocyanin was drastically reduced in atugt79b1 knockout mutants [49], which may explain elevated ROS accumulation in NbUGT91C1-silenced leaves (Figure 4C). We further found that overexpression of NbUGT74F1 or NbUGT91C1 promoted TuMV replication (Figure 5). Thus, we believe that TuMV can upregulate these two UGTs levels to modify SA and anthocyanin levels, suppressing ROS accumulation, and finally benefit viral infection.

## 5. Conclusions

We employed the iTRAQ-based quantitative proteomics approach to determine the proteomes of TuMV and TuMV-△GDD-infected plants to compare proteins involved in viral replication and host defense pathways. We identified several DAPs, validated four DAPs belonging to the UGT family and further characterized their positive role in TuMV infection. This work provided important information on additional host proteins involved in TuMV replication and defense, and it shed new insights into the role of UGTs in viral infection.

## Data Availability

All data in this study is available in the main text.

## Supplementary Materials

The following supporting information can be downloaded at: https://www.mdpi.com/article/10.3390/v15061401/s1, Table S1. List of primers used in this study; Table S2. List of DAPs under TuMV-GFP and TuMV-GFP//△GDD infection in *N. benthamiana*; Figure S1. KOG functional classification of differentially accumulated proteins grouped into three major categories—biological process, cellular component and molecular function; Figure S2. Validation of the TRV-based gene silencing efficiency by RT-qPCR; Figure S3. Effect of transient silencing of NbUGTs on TuMV infection. (A) GFP fluorescence in *N. benthamiana* plants inoculated with TuMV-GFP together with ds:GUS (control), ds:NbUGT74F1, or ds:NbUGT91C1. (B) RT-qPCR analysis of the gene silencing efficiency and TuMV RNA level at 3 dpai. (C) Western blotting results of TuMV CP accumulation in ds:GUS (control)-, ds:NbUGT74F1-, and ds:NbUGT91C1-treated regions at 3dpai.

## References

[1] Vélez-Bermúdez I.C., Wen T.-N., Lan P., Schmidt W., Lois L.M., Matthiesen R. (2016). Isobaric tag for relative and absolute Quantitation (iTRAQ)-Based Protein Profiling in Plants. Plant Proteostasis: Methods and Protocols.

[2] Bowles D., Isayenkova J., Lim E.K., Poppenberger B. (2005). Glycosyltransferases: Managers of small molecules. Curr. Opin. Plant Biol..

[3] Bowles D., Lim E.-K. (2010). Glycosyltransferases of small molecules: Their roles in plant biology. Encyclopedia of Life Sciences.

[4] Wilson A.E., Tian L. (2019). Phylogenomic analysis of UDP-dependent glycosyltransferases provides insights into the evolutionary landscape of glycosylation in plant metabolism. Plant J..

[5] Dong N.Q., Sun Y., Guo T., Shi C.L., Zhang Y.M., Kan Y., Xiang Y.H., Zhang H., Yang Y.B., Li Y.C. (2020). UDP-glucosyltransferase regulates grain size and abiotic stress tolerance associated with metabolic flux redirection in rice. Nat. Commun..

[6] Mateo-Bonmati E., Casanova-Saez R., Simura J., Ljung K. (2021). Broadening the roles of UDP-glycosyltransferases in auxin homeostasis and plant development. New. Phytol..

[7] Hu Y., Zhang M., Lu M., Wu Y., Jing T., Zhao M., Zhao Y., Feng Y., Wang J., Gao T. (2022). Salicylic acid carboxyl glucosyltransferase UGT87E7 regulates disease resistance in Camellia sinensis. Plant Physiol..

[8] He Y., Wu L., Liu X., Jiang P., Yu L., Qiu J., Wang G., Zhang X., Ma H. (2020). TaUGT6, a novel UDP-Glycosyltransferase gene enhances the resistance to FHB and DON accumulation in wheat. Front. Plant Sci..

[9] Li P., Li Y.J., Zhang F.J., Zhang G.Z., Jiang X.Y., Yu H.M., Hou B.K. (2017). The Arabidopsis UDP-glycosyltransferases UGT79B2 and UGT79B3, contribute to cold, salt and drought stress tolerance via modulating anthocyanin accumulation. Plant J..

[10] Liu Y., Wang Q., Liu X., Cheng J., Zhang L., Chu H., Wang R., Li H., Chang H., Ahmed N. (2023). pUGTdb: A comprehensive database of plant UDP-dependent glycosyltransferases. Mol. Plant.

[11] Wu J., Zhu W., Shan X., Liu J., Zhao L., Zhao Q. (2022). Glycoside-specific metabolomics combined with precursor isotopic labeling for characterizing plant glycosyltransferases. Mol. Plant.

[12] Yang X., Li Y., Wang A. (2021). Research advances in potyviruses: From the laboratory bench to the field. Annu. Rev. Phytopathol..

[13] Inoue-Nagata A.K., Jordan R., Kreuze J., Li F., López-Moya J.J., Mäkinen K., Ohshima K., Wylie S.J., ICTV Report Consortium (2022). ICTV virus taxonomy profile: Potyviridae 2022. J. Gen. Virol..

[14] Revers F., Garcia J.A. (2015). Molecular biology of potyviruses. Adv. Virus Res..

[15] Rodamilans B., Valli A., Garcia J.A. (2020). Molecular plant-plum pox virus interactions. Mol. Plant Microbe Interact..

[16] Martinez F., Carrasco J.L., Toft C., Hillung J., Gimenez-Santamarina S., Yenush L., Rodrigo G., Elena S.F. (2023). A binary interaction map between turnip mosaic virus and Arabidopsis thaliana proteomes. Commun. Biol..

[17] Nellist C.F., Ohshima K., Ponz F., Walsh J.A. (2022). Turnip mosaic virus, a virus for all seasons. Ann. Appl. Biol..

[18] Wu G.W., Cui X.Y., Chen H., Renaud J.B., Yu K.F., Chen X., Wang A.M. (2018). Dynamin-like proteins of endocytosis in plants are coopted by Potyviruses to enhance virus Infection. J. Virol..

[19] Wu G.W., Jia Z.X., Ding K.D., Zheng H.Y., Lu Y.W., Lin L., Peng J.J., Rao S.F., Wang A.M., Chen J.P. (2022). Turnip mosaic virus co-opts the vacuolar sorting receptor VSR4 to promote viral genome replication in plants by targeting viral replication vesicles to the endosome. PLoS Pathog..

[20] Cheng X., Xiong R., Li Y., Li F., Zhou X., Wang A. (2017). Sumoylation of turnip mosaic virus RNA polymerase promotes viral infection by counteracting the host NPR1-mediated immune response. Plant Cell.

[21] Ji M., Zhao J., Han K., Cui W., Wu X., Chen B., Lu Y., Peng J., Zheng H., Rao S. (2021). Turnip mosaic virus P1 suppresses JA biosynthesis by degrading cpSRP54 that delivers AOCs onto the thylakoid membrane to facilitate viral infection. PLoS Pathog..

[22] Cotton S., Grangeon R., Thivierge K., Mathieu I., Ide C., Wei T., Wang A., Laliberte J.F. (2009). Turnip mosaic virus RNA replication complex vesicles are mobile, align with microfilaments, and are each derived from a single viral genome. J. Virol..

[23] Shen W., Shi Y., Dai Z., Wang A. (2020). The RNA-dependent RNA polymerase NIb of potyviruses plays multifunctional, contrasting roles during viral infection. Viruses.

[24] Cui X.Y., Yaghmaiean H., Wu G.W., Wu X.Y., Chen X., Thorn G., Wang A.M. (2017). The C-terminal region of the Turnip mosaic virus P3 protein is essential for viral infection via targeting P3 to the viral replication complex. Virology.

[25] Le Roy J., Huss B., Creach A., Hawkins S., Neutelings G. (2016). Glycosylation is a major regulator of phenylpropanoid availability and biological activity in plants. Front. Plant Sci..

[26] Fernandez-Pozo N., Rosli H.G., Martin G.B., Mueller L.A. (2015). The SGN VIGS tool: User-friendly software to design virus-induced gene silencing (VIGS) constructs for functional genomics. Mol. Plant.

[27] Wang A. (2015). Dissecting the molecular network of virus-plant interactions: The complex roles of host factors. Annu. Rev. Phytopathol..

[28] Lum K.K., Cristea I.M. (2016). Proteomic approaches to uncovering virus–host protein interactions during the progression of viral infection. Expert Rev. Proteom..

[29] Yang X., Das P.P., Oppenheimer P., Zhou G., Wong S.M. (2020). iTRAQ-based protein analysis provides insight into heterologous superinfection exclusion with TMV-43A against CMV in tobacco (Nicotiana benthamiana) plants. J. Proteomics.

[30] Macharia M., Das P.P., Naqvi N.I., Wong S.-M. (2020). iTRAQ-based quantitative proteomics reveals a ferroptosis-like programmed cell death in plants infected by a highly virulent tobacco mosaic virus mutant 24A+UPD. Phytopathol. Res..

[31] Liu H., Das P.P., Zhang J., Yu L., Wang M., Lin Q., Zhou Y., Xu Q., Wong S.M. (2021). iTRAQ-based quantitative proteomics suggests mitophagy involvement after Rice black-streaked dwarf virus acquisition in insect vector small brown planthopper Laodelphax striatellus Fallen. J. Proteomics.

[32] Dodds P.N., Rathjen J.P. (2010). Plant immunity: Towards an integrated view of plant-pathogen interactions. Nat. Rev. Genet..

[33] Van Loon L.C., Rep M., Pieterse C.M. (2006). Significance of inducible defense-related proteins in infected plants. Annu. Rev. Phytopathol..

[34] Yun H.S., Kwon C. (2017). Vesicle trafficking in plant immunity. Curr. Opin. Plant Biol..

[35] Kaksonen M., Roux A. (2018). Mechanisms of clathrin-mediated endocytosis. Nat. Rev. Mol. Cell Biol..

[36] Wu G.W., Cui X.Y., Dai Z.J., He R.R., Li Y.Z., Yu K.F., Bernards M., Chen X., Wang A.M. (2020). A plant RNA virus hijacks endocytic proteins to establish its infection in plants. Plant J..

[37] Wu G.W., Jia Z.X., Rui P.H., Zheng H.Y., Lu Y.W., Lin L., Peng J.J., Rao S.F., Wang A.M., Chen J.P. (2022). Acidic dileucine motifs in the cylindrical inclusion protein of turnip mosaic virus are crucial for endosomal targeting and viral replication. Mol. Plant Pathol..

[38] He B., Cai Q., Qiao L., Huang C.Y., Wang S., Miao W., Ha T., Wang Y., Jin H. (2021). RNA-binding proteins contribute to small RNA loading in plant extracellular vesicles. Nat. Plants.

[39] Restrepo-Hartwig M.A., Carrington J.C. (1994). The tobacco etch potyvirus 6-kilodalton protein is membrane associated and involved in viral replication. J. Virol..

[40] Wei T., Zhang C., Hong J., Xiong R., Kasschau K.D., Zhou X., Carrington J.C., Wang A. (2010). Formation of complexes at plasmodesmata for potyvirus intercellular movement is mediated by the viral protein P3N-PIPO. PLoS Pathog..

[41] Movahed N., Cabanillas D.G., Wan J., Vali H., Laliberte J.F., Zheng H. (2019). Turnip mosaic virus components are released into the extracellular space by vesicles in infected leaves. Plant Physiol..

[42] Moller I.M., Sweetlove L.J. (2010). ROS signalling—Specificity is required. Trends Plant Sci..

[43] Peng Y., Yang J., Li X., Zhang Y. (2021). Salicylic acid: Biosynthesis and signaling. Annu. Rev. Plant Biol..

[44] Ding P., Ding Y. (2020). Stories of salicylic acid: A plant defense hormone. Trends Plant Sci..

[45] Huang X.X., Zhu G.Q., Liu Q., Chen L., Li Y.J., Hou B.K. (2018). Modulation of plant salicylic acid-associated immune responses via glycosylation of dihydroxybenzoic acids. Plant Physiol..

[46] Noutoshi Y., Okazaki M., Kida T., Nishina Y., Morishita Y., Ogawa T., Suzuki H., Shibata D., Jikumaru Y., Hanada A. (2012). Novel plant immune-priming compounds identified via high-throughput chemical screening target salicylic acid glucosyltransferases in Arabidopsis. Plant Cell.

[47] Dean J.V., Delaney S.P. (2008). Metabolism of salicylic acid in wild-type, ugt74f1 and ugt74f2 glucosyltransferase mutants of Arabidopsis thaliana. Physiol. Plant.

[48] George Thompson A.M., Iancu C.V., Neet K.E., Dean J.V., Choe J.Y. (2017). Differences in salicylic acid glucose conjugations by UGT74F1 and UGT74F2 from Arabidopsis thaliana. Sci. Rep..

[49] Yonekura-Sakakibara K., Fukushima A., Nakabayashi R., Hanada K., Matsuda F., Sugawara S., Inoue E., Kuromori T., Ito T., Shinozaki K. (2012). Two glycosyltransferases involved in anthocyanin modification delineated by transcriptome independent component analysis in Arabidopsis thaliana. Plant J..

[50] Landi M., Tattini M., Gould K.S. (2015). Multiple functional roles of anthocyanins in plant-environment interactions. Environ. Exp. Bot..

