# Supplementary material for: Proteomics Identified UDP-Glycosyltransferase Family Members as Pro-Viral Factors for Turnip Mosaic Virus Infection in Nicotiana benthamiana
Source: Viruses. 2023 Jun 20;15(6):1401. doi: 10.3390/v15061401 (PMC10301856; doi:10.3390/v15061401)
Supplement: Supplementary file 1 [file viruses-15-01401-s001.zip › viruses-2317066-supplementary/viruses-2317066-supplementary.pdf]

viruses

MDPI

Supplementary Materials

Proteomics Identified UDP-Glycosyltransferase Family Members as Pro-Viral Factors for Turnip Mosaic Virus Infection in *Nicotiana benthamiana*

Table S1. Primers used in this study.

Primer Names	Sequences (5′ to 3′)
Ptrv2-NbUGT73B5-LicF	gAggAgAagAgCCgTcgCACACAATAAAGATGTCAAGAC
Ptrv2-NbUGT73B5-LicR	CgACgACAAgACCgTCACCCTTGGAATTGAGCCAATCTAAAC
qNbUGT73B5-F	CTCGGCATGGTGTGAAGGCA
qNbUGT73B5-R	TTGCAAGGGAACTCGGTCGT
Ptrv2-UGT74F1-LicF	gAggAgAagAgCCgTcgATGGACTTGTTGAATAAC
Ptrv2-UGT74F1-LicR	CgACgACAAgACCgTCACCCTTGATAAGGTCCTCTAGGG
qNbUGT74F1-F	CACCTCCGGTGAGGATTCCG
qNbUGT74F1-R	GCGTCTACTACCTCAGCCTCCA
NbUGT74F1-licF	CgACgACAAgACCgTCACCATGGACTTGTTGAATAAC
NbUGT74F1-licR	gAggAgAagAgCCgTcgTTTAGTACATTTTGATAC
Ptrv2-UGT91C1-Lic-F	gAggAgAagAgCCgTcgATGGATACACAAGTAATAG
Ptrv2-UGT91C1-Lic-R	CgACgACAAgACCgTCACCTCTAAGGGCGTGATTGAGATG
qNbUGT91C1-F	TGCTAGGTTGATGGCAGAAC
qNbUGT91C1-R	CGTCTCTTCAGCCCTTACAG
NbUGT91C1-Lic-F	CgACgACAAgACCgTCACCATGGATACACAAGTAATAGAATG
NbUGT91C1-Lic-R	gAggAgAagAgCCgTcgTTTACACTTATTACTAGTCTTAC
Ptrv2NbUFGT3-lic-F	gAggAgAagAgCCgTcgCTGCCTTCATTTTGAGTCCCT
Ptrv2NbUFGT3-lic-R	CgACgACAAgACCgTCACCTTCGGCGAAGGTGTTGACCAT
qNbUFGT3-F	AGCTGGCCACCTCATCTCCA
qNbUFGT3-R	ACGAACTGTAAGCGGGCTGT
qNbActin2-F	GGGATGTGAAGGAGAAGTTGGC
qNbActin2-R	ATCAGCAATGCCCGGGAACA
ds*UGT74F1*-F1	ggcgcgccccatgcgggatcAGCTCTTCCTTATCCAAGCCA
ds*UGT74F1*-R1	catttggagaggacacgcggTGCGAGCCGATTTTCTTGAA
ds*UGT74F1*-F2	caatttgcaggtatttggctAGCTCTTCCTTATCCAAGCCA
ds*UGT74F1*-R2	ggtcttaattaactctctagTGCGAGCCGATTTTCTTGAA
ds*UGT91C1*-F1	ggcgcgccccatgcgggatcACTGATGTTTCCATGGTTAGCA
ds*UGT91C1*-R1	catttggagaggacacgcggGTGATTGAGATGGGGTGGGA
ds*UGT91C1*-F2	caatttgcaggtatttggctACTGATGTTTCCATGGTTAGCA
ds*UGT91C1*-R2	ggtcttaattaactctctagGTGATTGAGATGGGGTGGGA

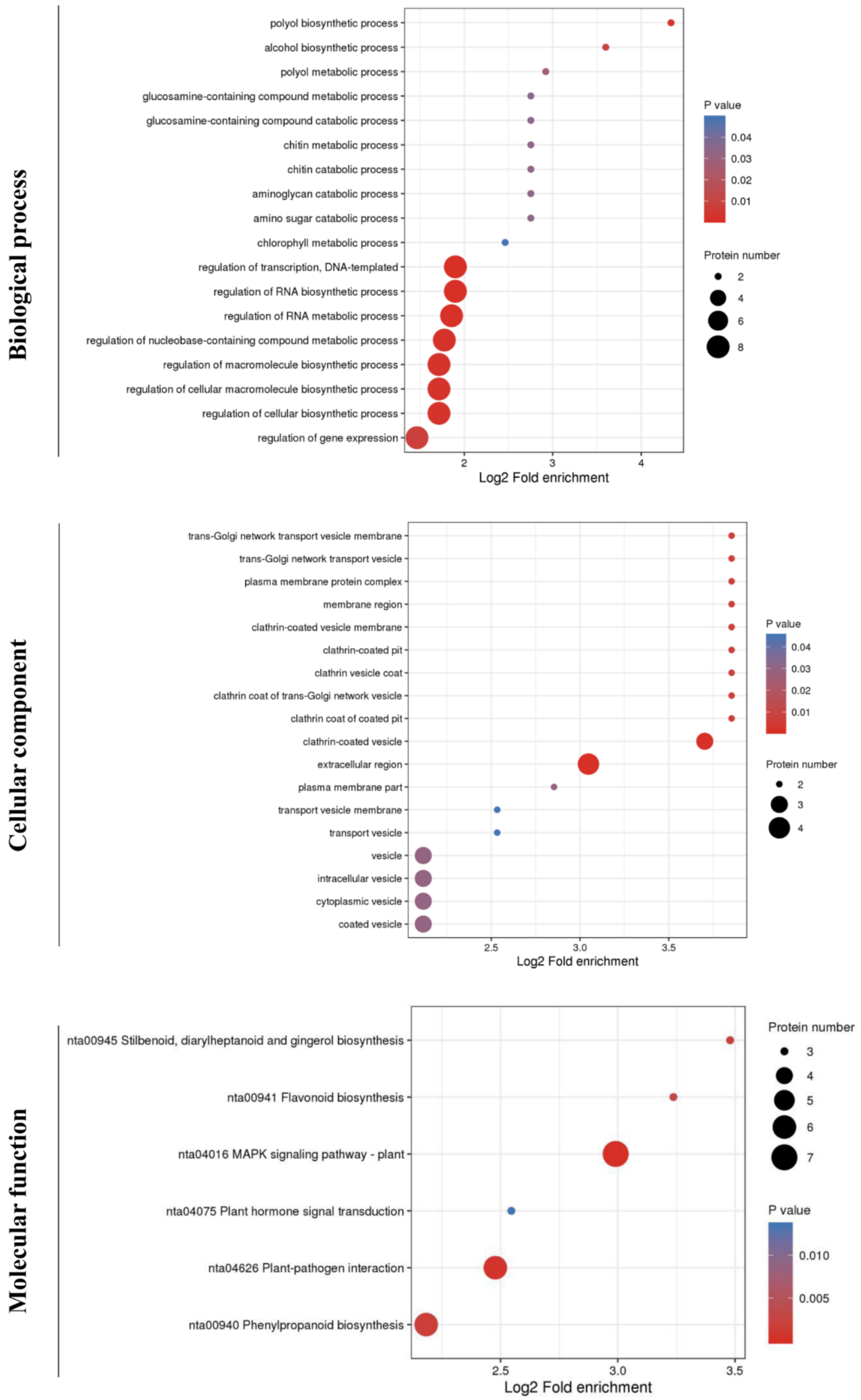

Figure S1. KOG functional classification of differentially accumulated proteins grouped into three major categories—biological process, cellular component and molecular function.

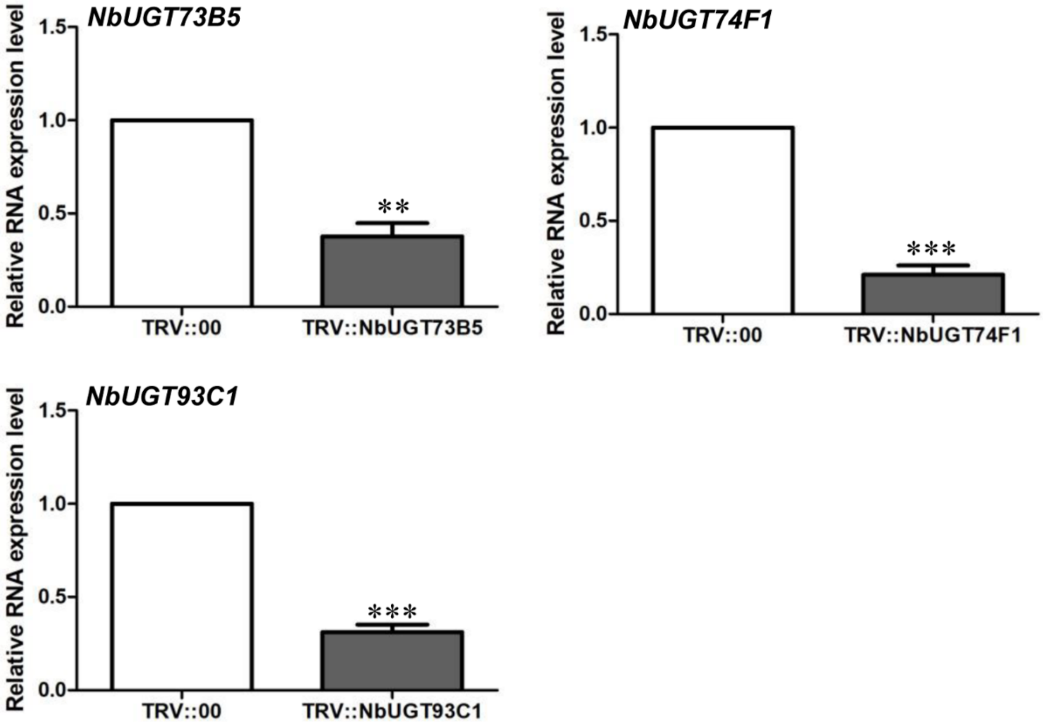

Figure S2. Validation of the TRV-based gene silencing efficiency by RT-qPCR. ** and *** represent statistically significant differences by the Student t-test between groups at $p < 0.01$ and $p < 0.001$, respectively.

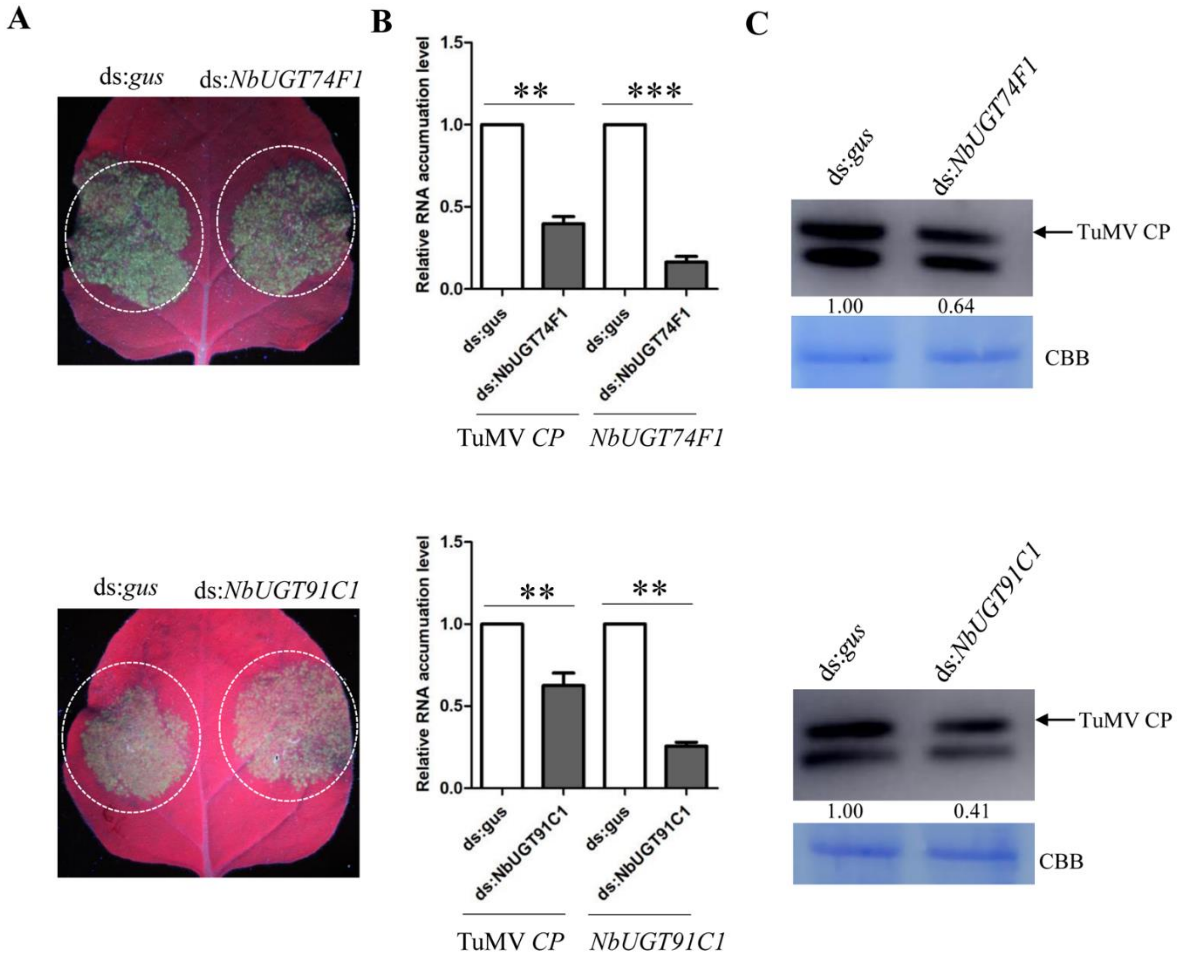

Figure S3. Effect of transient silencing of NbUGTs on TuMV infection. (**A**) GFP fluorescence in *N. benthamiana* plants inoculated with TuMV-GFP together with ds:GUS (control), ds:NbUGT74F1, or ds:NbUGT91C1. (**B**) RT-qPCR analysis of the gene silencing efficiency and TuMV RNA level at 3 dpai. (**C**) Western blotting results of TuMV CP accumulation in ds:GUS (control)-, ds:NbUGT74F1-, and ds:NbUGT91C1-treated regions at 3dpai. ** represent statistically significant differences by the Student *t*-test between groups at $p < 0.01$.
